# Potential mechanisms and prognostic model of eRNAs-regulated genes in stomach adenocarcinoma

**DOI:** 10.1038/s41598-022-20824-1

**Published:** 2022-10-03

**Authors:** Liuying Gao, Hao Rong

**Affiliations:** 1 The Affiliated People’s Hospital of Ningbo University, Ningbo, 315040 China; 2 Department of Preventive Medicine, Zhejiang Provincial Key Laboratory of Pathological and Physiological Technology, School of Medicine, Ningbo, 315211 China

**Keywords:** Cancer genetics, Gastrointestinal cancer

## Abstract

Gastric Carcinoma is the fourth leading cause of cancer deaths worldwide, in which stomach adenocarcinoma (STAD) is the most common histological type. A growing amount of evidence has suggested the importance of enhancer RNAs (eRNAs) in the cancer. However, the potential mechanism of eRNAs in STAD remains unclear. The eRNAs-regulated genes (eRRGs) were identified through four different enhancer resources. The differentially expressed eRRGs were obtained by ‘DESeq2’ R package. The prognosis prediction model was constructed by Cox and Lasso regression analysis. The ‘ChAMP’ R package and ‘maftools’ R package were used to investigate the multi-omics characters. In this study, combining the concept of contact domain, a total of 9014 eRRGs including 4926 PCGs and 4088 lncRNAs were identified and these eRRGs showed higher and more stable expression. Besides, the functions of these genes were mainly associated with tumor-related biological processes. Then, a prognostic prediction model was constructed and the AUC values of the 1-, 3- and 5-year survival prediction reached 0.76, 0.84 and 0.84, respectively, indicating that this model has a high accuracy. Finally, the difference between high-risk group and low-risk group were investigated using multi-omics data including gene expression, DNA methylation and somatic mutations. Our study provides significant clues for the elucidation of eRNAs in STAD and may help improve the overall survival for STAD patients.

## Introduction

Gastric Carcinoma (GC) is a malignant tumor arising from the gastric mucosal epithelium, and about 90% are stomach adenocarcinoma (STAD). GC ranks fifth in global cancer incidence and fourth in mortality. In 2020, over 1 million newly diagnosed cases and approximately 769,000 deaths are estimated to occur worldwide^1,2^. In 2015, the incidence of gastric cancer was approximately 29.31/100,000 and the mortality rate was 21.16/100,000 in China. Although the incidence and mortality of GC are decreasing in China, the number of patients and deaths is still high. In most countries, GC has low 5-year survival rate, with no more than 30%^3,4^. Therefore, identifying novel biomarkers is important to elucidating the mechanism of GC development.

Enhancers are DNA regulatory elements that can control the spatiotemporal specific expression of genes^5,6^. For example, in the ventral midbrain and posterior diencephalon, Sonic Hedgehog brain enhancer-1 (SBE1) can specifically enhance gene expression^7^. Recent studies have found that enhancers can transcribe long non-coding RNAs (lncRNAs), which are defined as enhancer RNAs (eRNAs)^8–11^. eRNAs can also participate in various cancer signaling pathways by regulating the expression of their target genes. For example, *KLK3* eRNA is involved in androgen receptor (AR)-dependent complex looping and selectively enhances the expression of gene *KLK3* in prostate cancer^12^. An increasing number of studies have shown that the activation of many oncogenes is usually accompanied by the enhancer activation and eRNA production^13^. The evidences mentioned above reveal the key role of eRNAs in understanding the functional mechanisms of cancer. However, there are few researches on the transcriptome analysis of eRNAs in STAD.

Here we identify eRNAs-regulated genes (eRRGs) in STAD. The eRNAs were obtained from the enhancer resources including Ensembl^14^, Functional Annotation of the Mammalian Genome (FANTOM)^15^, Roadmap Epigenomics^16^ and Encyclopedia of DNA Elements (ENCODE) project^17^. What’s more, previous study found that genomes are partitioned into contact domains (median length, 185 kb), which are related to gene activation, and show conservation across cell types and species^18^. So, we combined the concept of contact domain to identify the eRRGs. Then, the differentially expressed eRRGs (DEeRRGs) were calculated using expression profiles of STAD from The Cancer Genome Atlas (TCGA) portal^19^. We also constructed a prognosis prediction model using these DEeRRGs. Finally, the difference between high-risk group and low-risk group was investigated using multi-omics data including gene expression, DNA methylation and somatic mutations. Our results will provide significant clues for the elucidation of eRNAs in STAD.

## Materials and methods

### Identification of eRRGs and data acquisition

We collected the annotations of enhancers from Ensembl^14^, FANTOM^15^, Roadmap Epigenomics^16^ and ENCODE^17^. What’s more, the ENCODE and Roadmap databases considered monomethylation of histone H3 at lysine 4 (H3K4me1) and acetylation of histone H3 at lysine 27 (H3K27ac) marks in stomach tissues. All genome coordinates were converted to GRCh38 by LiftOver tool^20^. Annotations of genes were collected from GENCODE (release 36)^21^. The eRNA regions were defined as the ± 3 kb of the middle loci of enhancers^22,23^. Meanwhile, the eRNA that overlapped with known genes regions were filtered out. The PCGs and lncRNAs whose genome regions were in the contact domain (approximately 185-kb) of eRNAs regions were determined as eRRGs^18^. Firstly, the eRRGs were obtained based on every enhancer database and get the intersection of the above four resources. RNA-seq profiles and corresponding clinical data of 375 STAD samples and 32 normal control samples, DNA mutation data of 440 STAD samples, and 397 profiles of the Illumina 450 k DNA methylation array were collected from the TCGA portal.

### The expression changes of eRRGs

The eRRGs lacking expression values in all samples were filtered out. Then, ‘DESeq2’ R package^24^ were performed to determine the DEeRRGs between cancer samples and normal samples, and the significant level was served as false discovery rate (FDR) < 0.05 and the absolute of log2 fold change (FC) > 1. The heatmap of top20 up-regulated and top20 down-regulated DEeRRGs expression was generated by ‘pheatmap’ R package.

### Functional enrichment analysis

The GO analysis of the eRRGs was performed using ‘clusterProfiler’ R package^25^. The function enrichment of lncRNAs was conducted by online tools ncFANs v2.0^26^. The PPI network of DEeRRGs was obtained from STRING database^27^ and the Cytoscape was used to visualize the network^28^. The CytoHubba application was used to identify the remarkable nodes in PPI by MCC method^29^, and the MCODE algorithm was performed to find the important modules of PPI^30^.

### Establishing and evaluating the prognosis prediction model

The workflow to explore the eRRGs prognostic signatures in STAD consists of four steps: (1) univariate Cox regression analysis was used to assess the individual effect of every DEeRRGs and set *p* < 0.05 as significant level by the ‘survival’ R package; (2) Lasso analysis using “glmnet” function^31^ was adopted to filter out less informative DEeRRGs, and the tenfold cross-validation was used to prevent overfitting; (3) multivariate Cox regression with stepwise process were used to obtain a prognostic prediction model. In model construction, 326 STAD samples were randomly divided into three equal parts, of which each part took turns as the independent testing set, and the remaining two parts serve as the training set; (4) The final prognostic prediction model were established based on the whole cohort. And the risk score of each patient was predicted based on the final model by using the ‘predict’ function. Then, patients were divided into high-risk group and low-risk group according to the median risk score. The Kaplan–Meier method was employed to estimate OS of patients for each group. What’s more, the univariate and multivariate Cox regression were used to test the clinical value of risk score. Finally, we constructed the nomogram by Cox analysis.

### Multi-omics analysis between high-risk group and low-risk group

We aimed to investigate the differences between the high-risk group and low-risk group in gene expression, DNA methylation and somatic mutations. For gene expression, the DEGs between the high-risk group (n = 163) and low-risk group (n = 163) were identified by ‘DESeq2’ R package^24^, considering FDR < 0.05 and the absolute of log2 FC > 1 as the significant level. For DNA methylation, the ‘ChAMP’ R package^32^ was used to process the methylation array data. The missing values were imputed by the ‘Combine’ method and 305 samples were used including 150 high-risk samples and 155 low-risk samples. The beta values were normalized using peak-based correction (PBC). The DMPs between high-risk group and low-risk group were calculated by the ‘champ.DMP’ function, considering FDR < 0.05 and the absolute of Δβ > 0.15 as statistically significant cutoff. For somatic mutations, whole exon sequencing data using ‘Mutect2’ was obtained from TCGA portal including 162 high-risk samples and 162 low-risk samples. Fisher’s exact test was used to determine the differential mutation genes and types with *p* < 0.05 as the significant level. The mutually exclusive and co-occurring mutation patterns were identified by ‘somaticInteractions’ function in ‘maftools’ R package^33^.

### Method statement

All methods were carried out in accordance with the relevant guidelines and regulations.

## Results

### Identification of eRRGs for STAD

**Figure 1:**
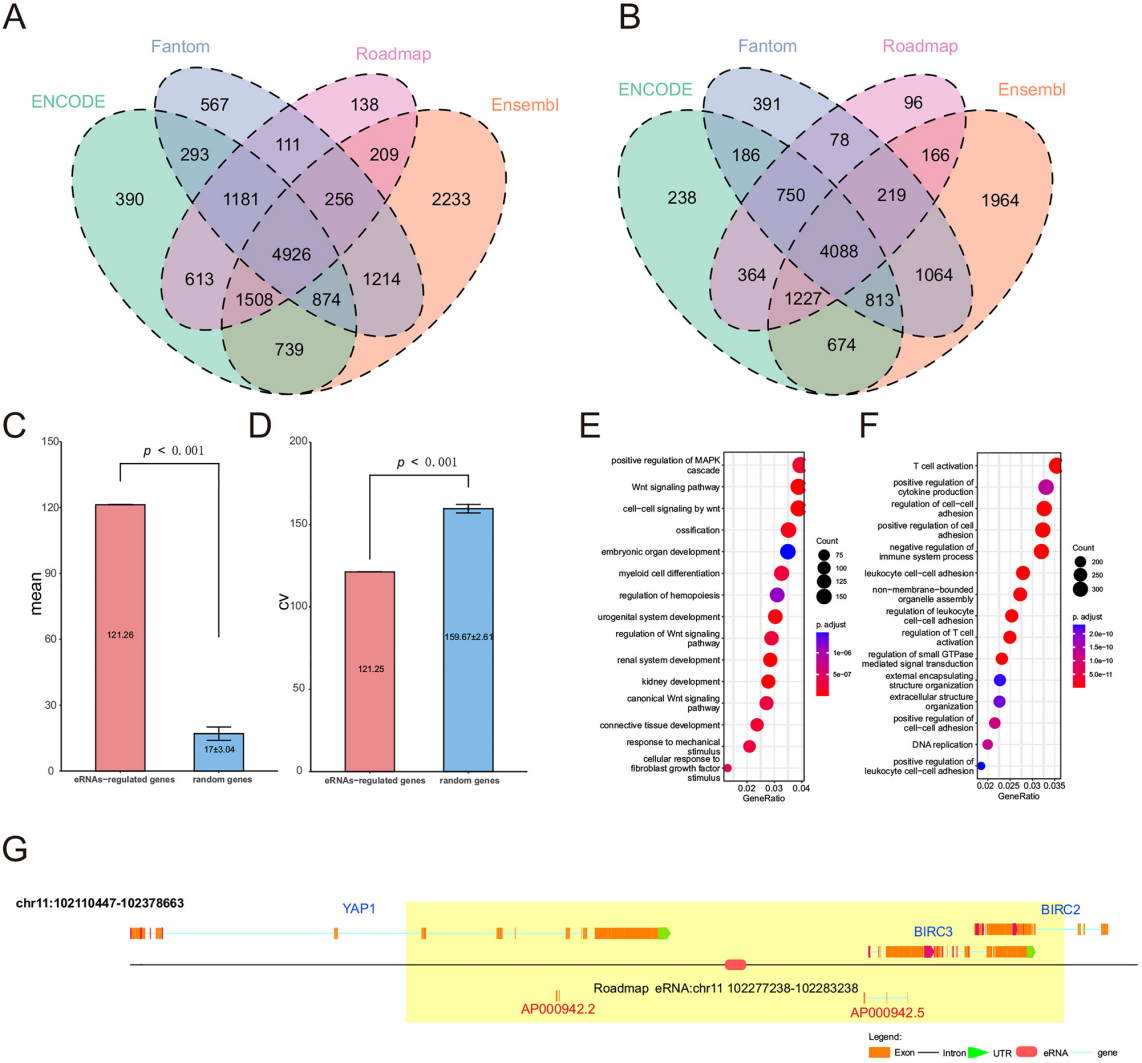
The characters of eRRGs. The numbers of eRNAs-regulated PCGs (**A**) and eRNAs-regulated lncRNAs (**B**) in Ensembl, Fantom, Roadmap and ENCODE database are shown in the Venn diagram. The barplots show the mean (**C**) and CV (**D**) of gene expression between eRRGs and random genes. Bubble plots show the results of functional enrichment analyses on eRNAs-regulated PCGs (**E**) and eRNAs-regulated lncRNAs (**F**). The color of the bubble represents the values of FDR and the size reflects the overlapped gene numbers between each GO term and query gene set. (**G**) The gene structure diagrams show the position of genes and eRNA in chromosome 11: 102277238–102283238. The PCGs are shown in blue and lncRNAs are shown in red. The yellow rectangle denotes the contact domains of eRNA.

We obtained enhancer annotations from Ensembl^14^, FANTOM^15^, Roadmap Epigenomics^16^ and ENCODE^17^ (Table S1). Given the fact that eRNAs regions could be wider than the enhancer ChIP-seq peaks, we defined the eRNA-transcribing regions as ± 3 kb of the middle loci of these annotated enhancers. After filtering out the eRNAs that overlapped with known genes, we obtained 36,499, 14,955, 22,425 and 27,448 eRNAs from Ensembl, FANTOM, Roadmap Epigenomics and ENCODE, respectively (Table S2). Then, we calculated the protein-coding genes (PCGs) and long non-coding RNAs (lncRNAs) whose genome regions were in the contact domain of eRNAs regions^18^. This analysis led to 22,174, 17,011, 15,930 and 18,864 eRRGs in Ensembl, FANTOM, Roadmap Epigenomics and ENCODE, respectively (Table S3). Finally, a total of 9014 eRRGs including 4926 PCGs and 4088 lncRNAs were obtained (Fig. 1A, B and Table S4).

In order to verify the expression characteristics of eRRGs, we randomly selected same number genes as random gene set and compared the mean and coefficients of variance (CV) of eRRGs to random gene set, and we found eRRGs had a higher expression than random genes (mean = 121.26 vs. 17.00, Wilcoxon rank-sum test, *p* < 0.001) (Fig. 1C). Meanwhile, the CV of eRRGs is lower than random genes (CV = 121.25 vs. 159.67, Wilcoxon rank-sum test, *p* < 0.001) (Fig. 1D).

Functional enrichment analysis showed these 9014 eRRGs tend to regulate the biological processes associated with cancer. For example, the top enriched gene ontology (GO) terms in PCGs were associated with MAPK and Wnt signaling pathway, and the abnormalities in these two pathways can affect cancer development^34–36^ (Fig. 1E). Meanwhile, the function of lncRNAs were mainly related to the immune system including T cell activation, positive regulation of cytokine production and negative regulation of immune system process (Fig. 1F).

Figure 1G showed the regulated-genes of Roadmap eRNA: chr11 102,277,238–102,283,238 including 3 PCGs and 2 lncRNAs. For example, the relationship between *YAP1* and eRNA has been reported in the literature^22^. Taken together, eRRGs could have the potential to be biomarkers and therapeutic target genes.

### Identification of differentially expressed eRRGs

**Figure 2:**
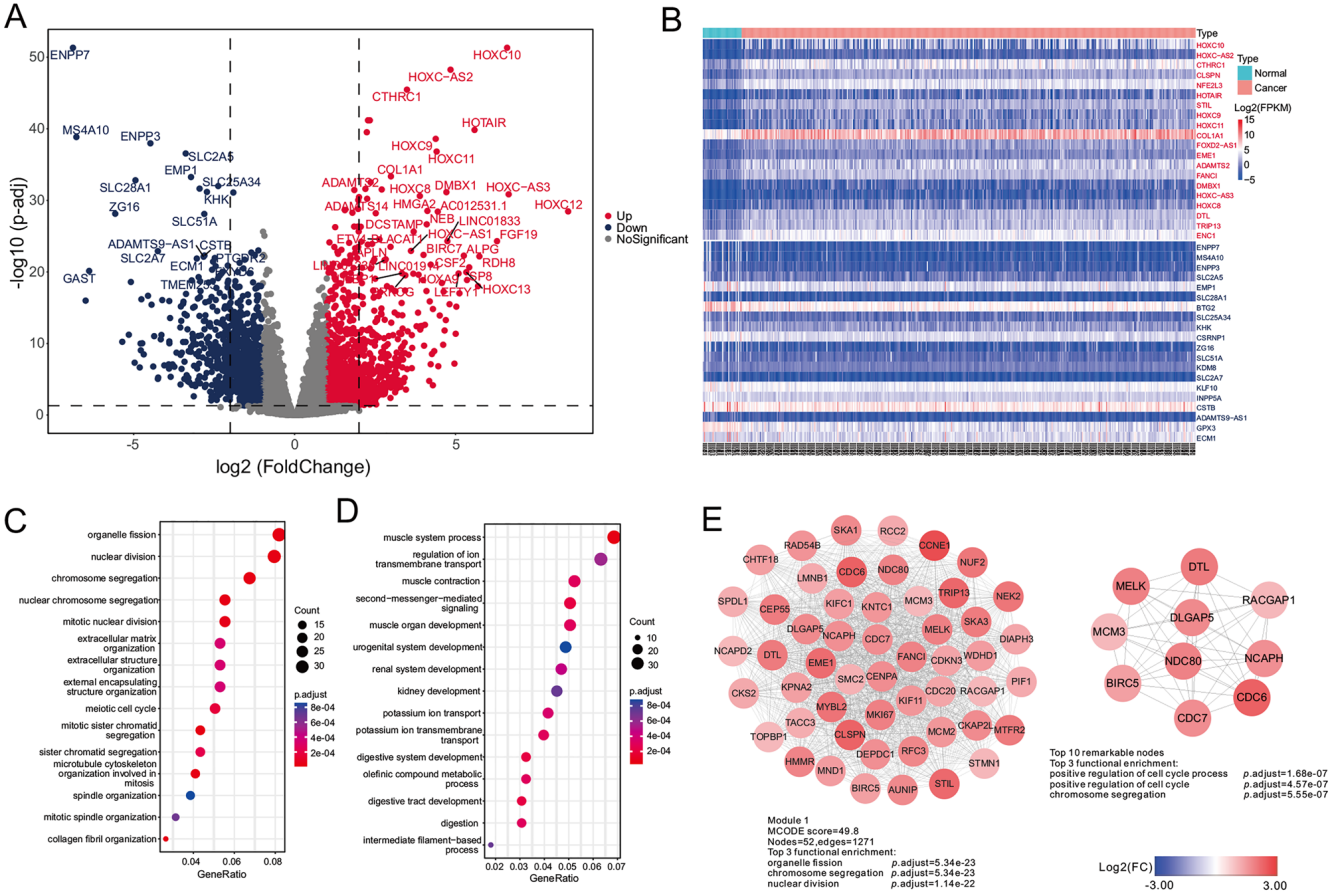
The expression changes of eRRGs between STAD samples and corresponding normal samples. (**A**) The differentially up-regulated eRRGs (red nodes) and down-regulated eRRGs (blue nodes) are shown in volcano plot. (**B**) The expression profiles of top 20 up-regulated eRRGs (red) and top 20 down-regulated eRRGs (blue) is shown in heatmap. The grouping information is shown in the top bar and the color represents the value of log_2_FPKM (fragments per kilobase of transcript per million mapped reads). The bubble plots show the functional enrichment analyses of up-regulated eRRGs (**C**) and down-regulated eRRGs (**D**). The size of bubble reflects the overlapped gene numbers between each GO term and query gene set. (**E**) The left plot shows the important module having the highest MCODE score and corresponding functional enrichment results. The right plot represents the top 10 remarkable nodes ranked by MCC and corresponding functional enrichment results. The color of nodes denotes the value of log_2_FC.

RNA-seq profiles of 375 STAD samples and 32 normal control samples from TCGA were used to identify the differential expressed eRRGs (DEeRRGs). A total of 2034 DEeRRGs were obtained, of which 1193 and 841 DEeRRGs, respectively, up-regulated and down-regulated in the STAD cohort (Fig. 2A, B, Table S5). What’s more, when using random gene sets as eRRGs, only 1069.90 up-regulated and 563.46 down-regulated genes were found on average (Figure S1). Note that several *HOXC* family members such as *HOXC10*, *HOXC9* and *HOXC11* were found to be significantly up-regulated (Fig. 2B), which have been experimentally verified to promoter the cell proliferation and migration in STAD^37–41^. Besides, the lncRNA *HOXC − AS2* and *HOXC − AS3* were also found to be up-regulated in STAD patients, which also can mediate tumorigenesis of STAD^42^.

To understand the potential functions of these two parts of DEeRRGs, we performed the functional enrichment analyses. As we expected, the top enriched GO terms in up-regulated genes were association with cell division process such as organelle fission, nuclear division and chromosome segregation, hinting that the up-regulated DEeRRGs may be promoter the cancer cell proliferation (Fig. 2C). Alternatively, the down-regulated genes were mainly related to the muscle system process, muscle organ development and muscle contraction (Fig. 2D). The loss of muscle, one of the complications in cancer patients, was found to be associated with poorer survival and increased chemotherapy toxicity^43^.

What’s more, we constructed the protein–protein interaction (PPI) network of DEeRRGs based on the STRING database which including 1042 nodes and 27,406 edges (Figure S2 and Table S6). In line with the functional enrichment analyses, the top 10 remarkable nodes ranked by Maximal Clique Centrality (MCC) were also involved in the cell division processes like positive regulation of cell cycle and chromosome segregation (Fig. 2E). The important modules were extracted via molecular complex detection (MCODE) application. We found that the Module 1 is comprised of 52 up-regulated genes associated with cell division (Fig. 2E). Taken together, DEeRRGs may affect the patients' prognosis by cell division.

### Establishing and evaluating the prognosis prediction model

To identify the eRRGs-related prognostic signatures for STAD, a four-step workflow based on Cox regression and lasso regression was adopted. Additionally, in order to evaluate the robustness and validity of the model, we randomly divided STAD samples into the independent testing set and training set. Each third of the sample took turns as the testing set, thus constructing three corresponding prediction model. In the results (Table 1 and Figure S3), the average the area under the ROC curve (AUC) values of 1-, 3-, 5-year survival prediction on training sets reached 0.76, 0.78 and 0.77, respectively. With regards to the prediction performance on the testing sets, the average AUC values of 1-, 3-, 5-year survival prediction equal to 0.73, 0.72 and 0.77. Moreover, the samples were classified into high-risk group and low-risk group according to the median risk score in each set. As expected, high-risk group exhibited worse overall survival (OS) than the low-risk group in each set (Figure S3).

**Table 1:** The AUC values of three trained models.

Index	Model 1		Model 2		Model 3		Average	
	Training set 1	Test set 1	Training set 2	Test set 2	Training set 3	Test set 3	Training set	Test set
1-year AUC	0.76	0.71	0.73	0.73	0.80	0.75	0.76	0.73
3-year AUC	0.79	0.80	0.75	0.74	0.81	0.61	0.78	0.72
5-year AUC	0.86	0.78	0.73	0.92	0.71	0.62	0.77	0.77

**Figure 3:**
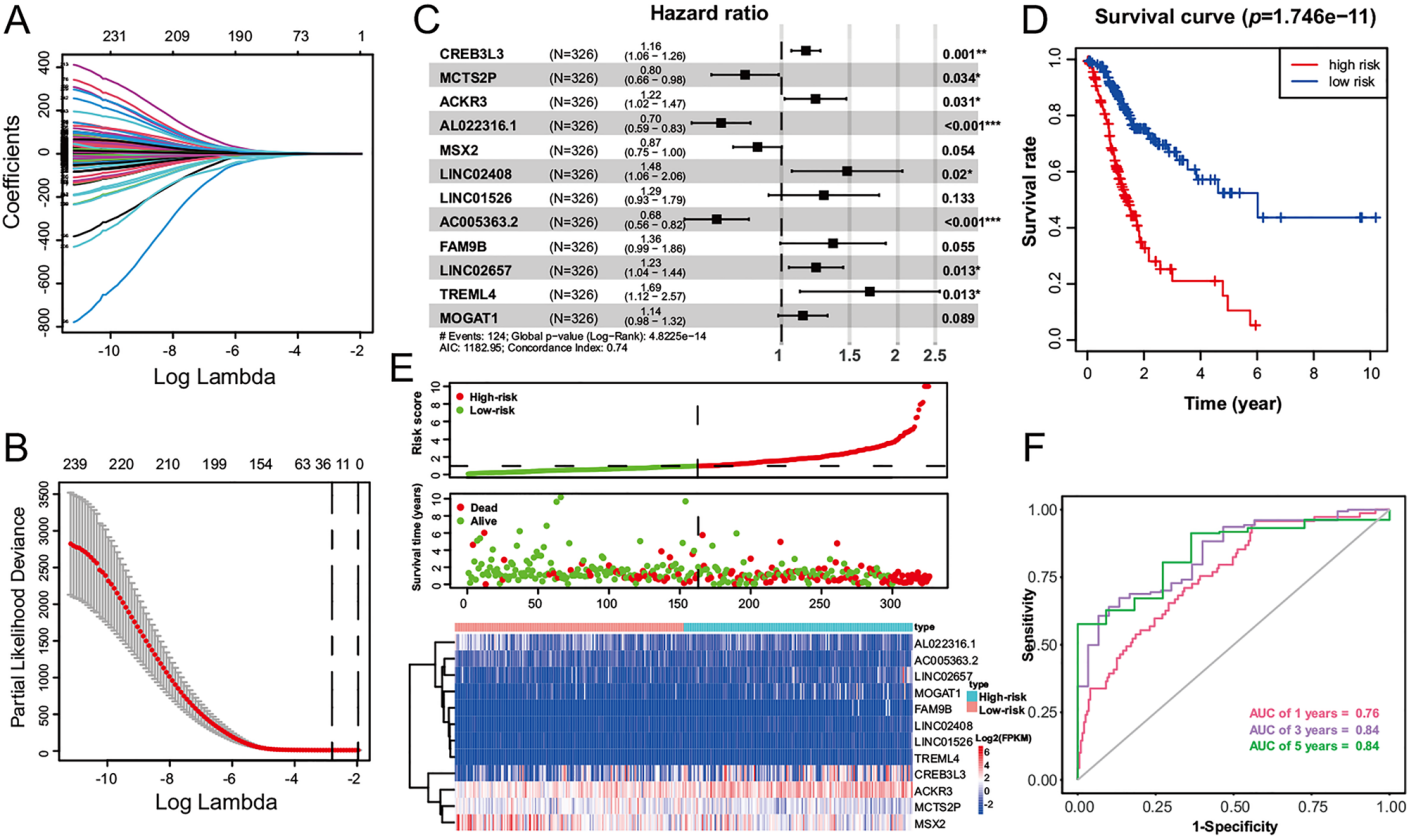
Constructing a prognostic prediction model for STAD. (**A**) The coefficients of lasso regression over different penalty parameter. (**B**) The optimal parameter lambda in lasso regression model. (**C**) Forest plot of the 12 prognostics related DEeRRGs. (**D**) KM curves compare the difference of OS between the high-risk group and low-risk group. (**E**) The distinct distributions of risk score (top panel), survival status (middle panel) and expression heatmap of 12 prognostics (bottom panel) between high-risk group and low-risk group. The color represents the value of log2FPKM. (**F**) The ROC curve of the risk score for predicting 1-, 3-, 5-year survival.

Considering the great robustness and validity of the prediction models, we then combined all STAD samples and constructed an overall model. After performing the univariate Cox regression, a total of 246 DEeRRGs were identified to have a significantly individual effect on the OS. In order to filter out the less contributive genes, we used lasso regression on these 246 DEeRRGs. Under the optimal parameter determined by tenfold cross-validation, 22 DEeRRGs were reserved (Fig. 3A, B) and used to establish the multivariate Cox regression model by stepwise method. The overall prediction model comprising of 12 DEeRRGs including *CREB3L3*, *MCTS2P*, *ACKR3*, *MSX2*, *FAM9B*, *TREML4*, *MOGAT1*, *AL022316.1*, *LINC02408*, *LINC01526*, *AC005363.2*, *LINC02657* (Fig. 3C). For example, the function of *MSX2* was found to affect the development of several human cancers such as osteosarcoma, breast cancer and pancreatic cancer^44–46^. Besides, the overexpression of *MSX2* promoters the cell proliferation and invasion, and attenuated cell cycle arrest and apoptosis^47^.

Moreover, in line with the above findings, the prognosis of the high-risk group is poorer compared to the low-risk group by log-rank test (*p* = 1.746*10^–11^, Fig. 3D). Moreover, the patients were divided into high-risk group and low-risk group according to the median of risk score, and high-risk group had more deaths and worse survival time than low-risk group (Fig. 3E). What’s more, the AUC values of the 1-, 3- and 5-year survival prediction reached 0.76, 0.84 and 0.84, respectively, showing high accuracy of the model (Fig. 3F).

### Evaluation of the clinical value of risk score

**Figure 4:**
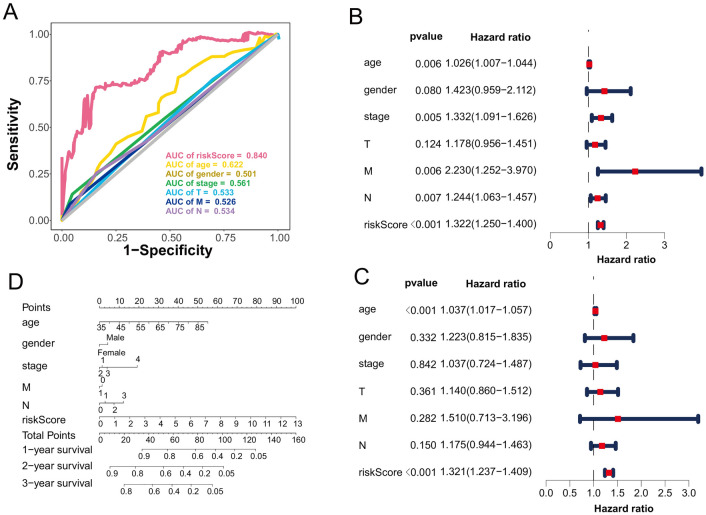
Identified the clinical value of risk score. (**A**) The AUC value of risk score and other clinical factors. The table shows the HR of risk score and other clinical factors calculated by univariate Cox regression (**B**) and multivariate Cox regression (**C**). (**D**) The nomogram constructed by different clinical factors in STAD patients.

Firstly, we assessed the diagnostic value of risk score and other clinical features by univariate Cox regression. Note that the AUC value of risk score reached 0.84 for 3-year survival is higher than other factors such as the AUC values of age, gender and stage only reached 0.622, 0.501 and 0.561, respectively (Fig. 4A).

In order to verify the clinical value of risk score, we calculated the hazard ratio (HR) of risk score and other clinical features using univariate and multivariate Cox regression. In the results (Fig. 4B, C), the HR of risk score equal to 1.322 (95% CI 1.250–1.400, *p* < 0.001) and 1.321 (95% CI 1.237–1.409, *p* < 0.001), indicating that the risk score has great clinical value for STAD patients. Besides, in the nomogram (Figs. 4D, S4), the risk score had the highest weighted point, and we can use this standard to predict the 1-, 2- and 3-year survival time for each patient.

### Identification of differentially expressed genes between high-risk group and low-risk group

**Figure 5:**
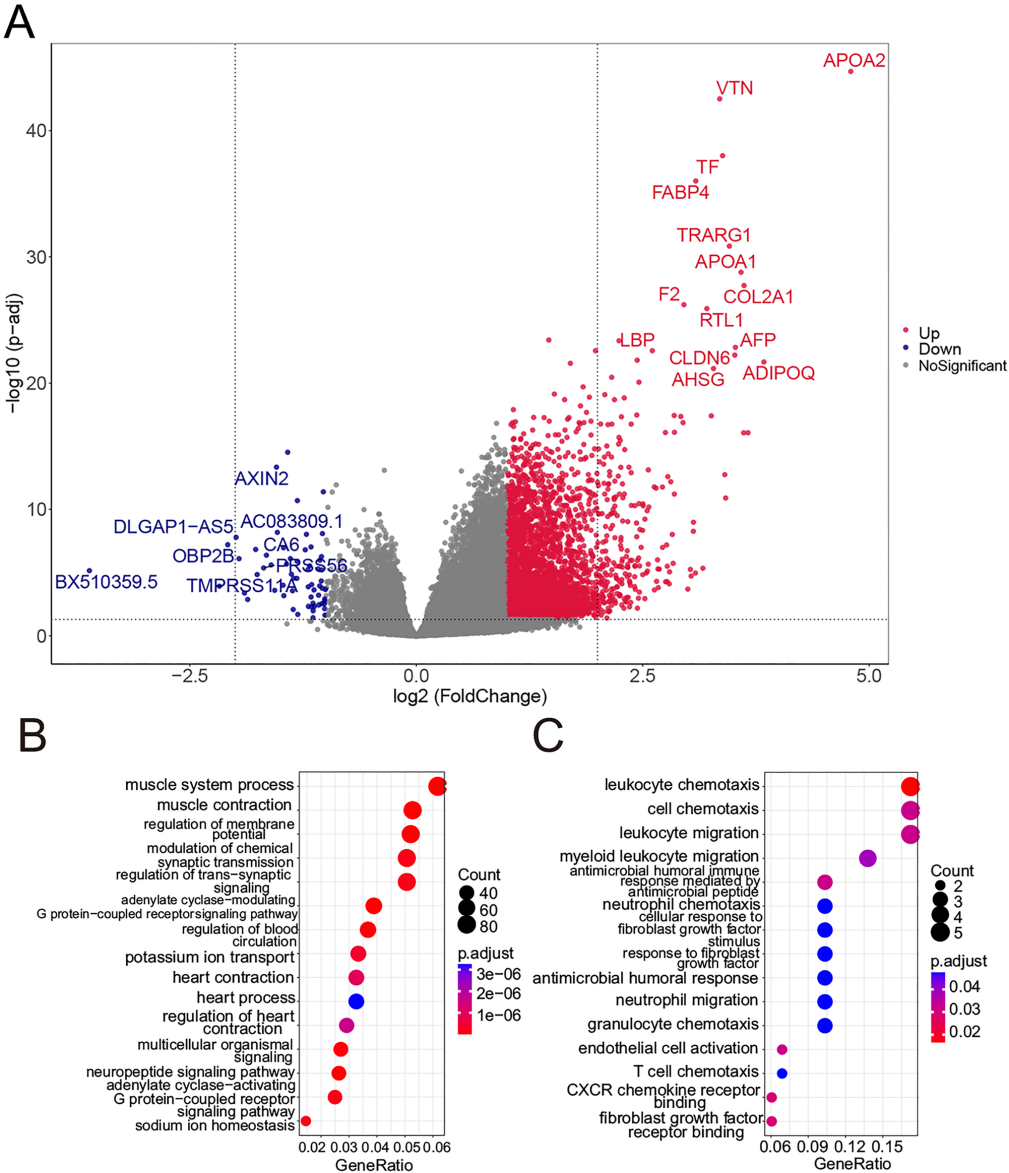
The distinct patterns of gene expression between high-risk group and low-risk group. (**A**) Volcano plot shows the differentially up-regulated genes (red nodes) and down-regulated genes (blue nodes). The bubble plots show the functional enrichment analyses of up-regulated genes (**B**) and down-regulated genes (**C**).

The expression profiles were used to identify the different expression pattern between high-risk group (n = 163) and low-risk group (n = 163). According to the standard of FDR < 0.05 and the absolute of log2 FC > 1, a total of 4001 differentially expressed genes (DEGs) were obtained, of which 3939 up-regulated and 62 down-regulated DEGs in high-risk group (Fig. 5A and Table S7). The function of the up-regulated genes was mainly associated with the biological processes like muscle contraction, regulation of membrane potential and regulation of trans-synaptic signaling (Fig. 5B). Furthermore, abnormal membrane potential plays an important role in tumor growth^48,49^. Besides, the muscle function will also influence the effect of cancer treatment^50,51^. Alternatively, the down-regulated genes were mainly enriched in the immune process such as leukocyte chemotaxis, leukocyte migration and myeloid leukocyte migration (Fig. 5C). Low immune status of high-risk group may lead to the poorer prognosis.

### Depicting DNA methylation pattern between high-risk group and low-risk group

**Figure 6:**
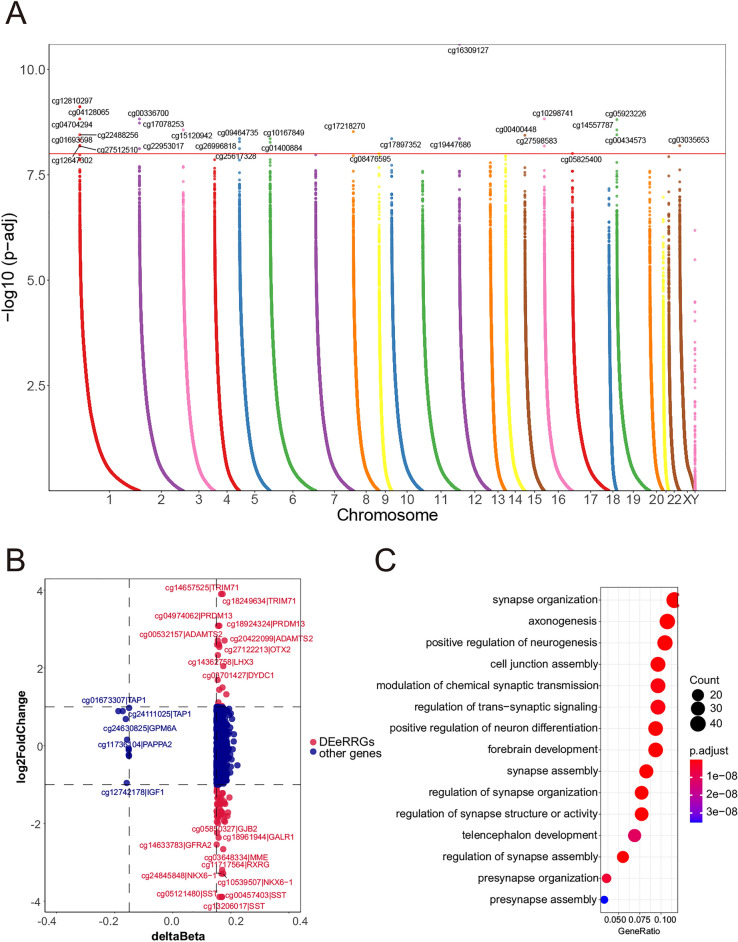
The difference of DNA methylation between high-risk group and low-risk group. (**A**) Manhattan plot of the differentially methylated probes in high-risk group and low-risk group. (**B**) The relationship between expression of eRRGs and DNA methylation level. The red nodes denote the DEeRRGs with Δβ > 0.15. (**C**) The bubble plots show the functional enrichment analyses of DMP-associated genes.

DNA methylation is a kind of important biological process associated with cancer development^52,53^. Hence, we investigated the differences of DNA methylation patterns between high-risk group (n = 150) and low-risk group (n = 155). In this process, a total of 907 prognostic-related differentially methylated probes (DMPs) were identified according to the standard of FDR < 0.05 and beta-value difference (Δβ) > 0.15 (Fig. 6A and Table S8). Compared with the low-risk group, 896 (98.79%) hypomethylated positions involving 424 genes were detected in the high-risk group. In contrast, the hypermethylated positions were only 11 (1.21%) related to 7 genes. Therefore, the high-risk group tends to have hypomethylated positions overall, but hypermethylated positions only occurs in a few genes.

What’s more, many DMP-associated genes were found to belong to DEeRRGs. Out of 424 hypomethylated genes in high-risk group, there were 12 up-regulated eRRGs and 27 down-regulated eRRGs (Fig. 6B). However, no DEeRRGs were found in hypermethylated genes. The function of DMP-associated genes was investigated based on functional enrichment analyses. Top 15 enriched GO terms were mainly related to tumor-associated neural processed such as synapse organization, axonogenesis and positive regulation of neurogenesis, indicating that the aberrant methylations in high-risk group may be through the recognition of neural pathways to affect the tumor development (Fig. 6C).

### The different somatic mutations patterns between high-risk group and low-risk group

**Figure 7:**
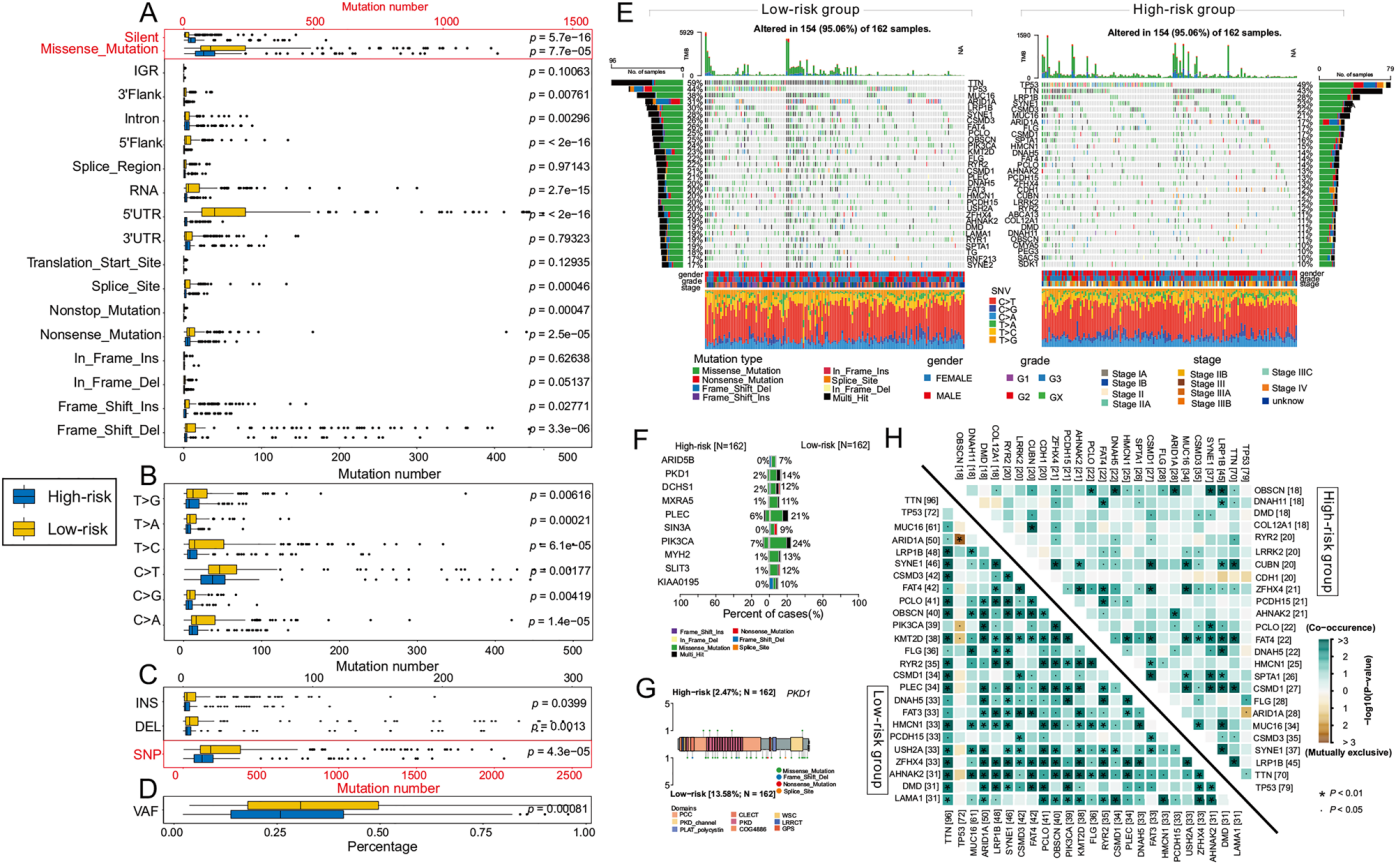
Landscape of somatic mutation in high-risk group and low-risk group. Boxplots show the comparisons of mutation numbers of mutation type (**A**), SNV (**B**), INDEL and SNP (**C**), and the percentage of VAF (**D**) between high-risk group and low-risk group. (**E**) Waterfall plot shows the mutation distribution of the top 25 most frequently mutated genes in high-risk group and low-risk group. The central panel shows the mutation type in each STAD sample. The lower part shows the clinical features (gender, grade and stage) and SNV types of each sample. The bottom panel denote the legend for mutation types and clinical features. (**F**) Forest plot displays the top 10 differentially mutated genes between two groups. (**G**) The lollipop plot illustrates the different mutation pattern in *PKD1*. (**H**) The co-occurring and exclusive mutations of the top 25 most frequently mutated genes is illustrated in the heatmap.

We further investigated the disparity of somatic mutations including the single-nucleotide variant (SNV), single-nucleotide polymorphism (SNP), insertion (INS), and deletion (DEL) between high-risk group (n = 162) and low-risk group (n = 162). In the results, most variants were missense mutations in both groups, and the low-risk group hold a significantly larger number of missense mutations (median = 110.00, interquartile range = 242.50) than those in the high-risk group (median = 76.00, interquartile range = 74.75) (Wilcoxon rank-sum test, *p* < 0.001) (Fig. 7A). As for SNV, a total of 24,516 and 61,732 SNVs were detected in high-risk group and low-risk group, respectively, of which C > T was the most common type in both high-risk group (median = 37, median proportion = 47.23%) and low-risk group (median = 51.5, median proportion = 50.00%). No matter the type of SNV, the low-risk group were significantly higher than those in the high-risk group (Wilcoxon rank-sum test, *p* < 0.05, Fig. 7B). Besides, the number of SNPs, INSs, DELs and VAF was also higher in low-risk group than those in high-risk group (Fig. 7C, D).

The top 15 most frequently mutated genes in corresponding groups were showed in Fig. 7E. As expect, most genes were participating in various tumor-associated biological processes in STAD like *TP53*, *TTN* and *LRP1B*^54–56^. Then, the differentially mutated frequencies were analyzed between the high-risk group and low-risk group, and 928 differentially mutated genes were obtained by Fisher’s exact test (*p* < 0.05). Figure 7F showed the top 10 differentially mutated genes. For example, *PKD1* plays a crucial role in gastric cancer cell migration and invasion^57^. And the different mutation of *PKD1* in high-risk group and low-risk group may be the reason for the poor prognosis of STAD patients (Fig. 7G). Finally, we investigated the co-occurring and exclusive mutations of the top 25 most frequently mutated genes. In the low-risk group, there were more co-occurring mutated pairs. The *ARID1A*-*TP53* was exhibiting mutually exclusive mutations in both groups, besides there were two other exclusive mutated pairs (*PIK3CA*-*TP53* and *KMT2D*-*TP53*) in low-risk group (Fig. 7H). This may be another reason for the difference between two groups.

## Discussion

eRNAs, transcribed by enhancers, are increasingly realized to affect the human cancer process by influencing gene transcription. In present study, we combined the concept of contact domain^18^ to identify the eRRGs and used four different enhancer annotation databases to reduce the false positive rate. As expect, these eRRGs show higher and more stable expression. And these genes were mainly associated with tumor-related biological processes like Wnt and MAPK signaling pathway. Note that the these eRRGs were also able to modulate the canonical immune biological processes such as T cell activation and positive regulation of cytokine production. These results indicate that eRRGs may have a crucial role in human cancers. Then, we analyzed the differentially expressed eRRGs in STAD samples. The up-regulated genes were mainly related to cell division process. And some up-regulated genes have been experimentally verified to promoter the cell proliferation and migration in STAD such as *HOXC* family members. We further constructed the prognostic prediction model and the AUC values of the 1-, 3- and 5-year survival prediction reached 0.76, 0.84 and 0.84, respectively, indicating that this model has a high accuracy. Besides, the risk score calculated by prediction model has a great clinical value and is a good independent prognostic factor for STAD patients. Finally, we used multi-omics data to investigate the differences between high-risk group and low-risk group. For gene expression, a total of 4001 DEGs were identified. Interestingly, the number of the down-regulated genes is only 62, but the function is mainly related to the immune process such as leukocyte chemotaxis, hinting that low immune status of high-risk group may lead to the poorer prognosis. For DNA methylation, we obtained 907 DMPs involving 431 genes. Note that the high-risk group tends to have more hypomethylated positions. The function of DMP-associated genes is mainly associated with tumor-associated neural processes like synapse organization. This difference may influence the patient’s prognosis. For somatic mutations, in line with the previous studies^58^, low-risk group shows higher mutation levels than high-risk group and might benefit from the immunotherapy. Additionally, there are two distinct exclusive mutated pairs (*PIK3CA*-*TP53* and *KMT2D*-*TP53*) in low-risk group. Therefore, we have strong reason to believe that the different mutation patterns between high-risk group and low-risk group have a crucial impact on the outcomes of STAD patients.

There are a few similar studies have been performed. For example, Gu et al.^59^ identified the key eRNAs and their regulated genes in squamous cell carcinoma of the head and neck. However, this study did not consider the tissue specificity of eRNA and only used the eRNA identified from the ENCODE database. Besides, Rong et al.^60^ screened the potential key eRNA-related genes in colon cancer. But, they did not filter the eRNA that overlapped with known genes regions. In this study, we not only considered these two issues, but also combined the concept of contact domain.

Although our study provides significant clues for the elucidation of eRNAs in STAD research, there are still some drawbacks that require further considered. First, the findings need further verification in Chinese because the ethnic in TCGA portal are mainly Europeans and Americans. Second, the somatic mutations data of lncRNAs is lacking because the MAF files in TCGA portal are mainly whole-exome sequencing data. Yet, despite such limitations, here is no denying that our study provides significant clues for the elucidation of eRNAs in STAD and might improve the OS for STAD patients.

## Conclusions

We totally obtained 9014 eRRGs including 4926 PCGs and 4088 lncRNAs in STAD, and the functions of these genes were mainly associated with tumor-related biological processes like Wnt and MAPK signaling pathway. Then, a prognostic prediction model was constructed and the AUC values of the 1-, 3- and 5-year survival prediction reached 0.76, 0.84 and 0.84, respectively, indicating that this model has a high accuracy. Finally, the difference between high-risk group and low-risk group were investigated using multi-omics data including gene expression, DNA methylation and somatic mutations. Our study provides significant clues for the elucidation of eRNAs in STAD and may help improve the overall survival for STAD patients.

## Supplementary Information

Supplementary Information 1.

Supplementary Information 2.

Supplementary Information 3.

Supplementary Information 4.

Supplementary Information 5.

Supplementary Information 6.

Supplementary Information 7.

Supplementary Information 8.

## Data Availability

The data that support the findings of this study are available in TCGA portal at https://portal.gdc.cancer.gov/; ENCODE and Roadmap project at https://www.encodeproject.org/; FANTOM at https://fantom.gsc.riken.jp/; Ensembl at https://asia.ensembl.org/index.html.

## Abbreviations

AUC: The area under the ROC curve
CV: Coefficients of variance
DEeRRGs: Differentially expressed eRRGs
DEL: Deletion
DMPs: Differentially methylated probes
ENCODE: Encyclopedia of DNA Elements
eRRGs: ERNAs-regulated genes
eRNAs: Enhancer RNAs
FANTOM: Functional Annotation of the Mammalian Genome
FC: Fold change
FDR: False discovery rate
GC: Gastric Carcinoma
GO: Gene ontology
H3K27ac: Acetylation of histone H3 at lysine 27
H3K4me1: Monomethylation of histone H3 at lysine 4
HR: Hazard ratio
INS: Insertion
lncRNAs: Long non-coding RNAs
MCC: Maximal Clique Centrality
MCODE: Molecular complex detection
OS: Overall survival
PBC: Peak-based correction
PCGs: Protein-coding genes
PPI: Protein–protein interaction
SBE1: Shh brain enhancer-1
Shh: Sonic Hedgehog
SNP: Single-nucleotide polymorphism
SNV: Single-nucleotide variant
STAD: Stomach adenocarcinoma
TCGA: The Cancer Genome Atlas
