# Supplementary material for: Potential mechanisms and prognostic model of eRNAs-regulated genes in stomach adenocarcinoma
Source: Sci Rep. 2022 Oct 3;12:16545. doi: 10.1038/s41598-022-20824-1 (PMC9529949; doi:10.1038/s41598-022-20824-1)
Supplement: Supplementary file 1 — Supplementary Information 1. [file 41598_2022_20824_MOESM1_ESM.pdf]

Supporting information

Table S1. The information of the enhancer annotation from Roadmap Epigenomics and ENCODE.

Table S2. The eRNAs identified from Ensembl, FANTOM, Roadmap Epigenomics and ENCODE.

Table S3. The eRRGs obtained from Ensembl, FANTOM, Roadmap Epigenomics and ENCODE.

Table S4. The list of final eRRGs.

Table S5. The list of the DEeRRGs.

Table S6. The information of the PPI network.

Table S7. The list of the differentially expressed genes between high-risk group and low-risk group.

Table S8. The information of the prognostic-related DMPs.

Figure S1. Comparison of the number of differentially expressed genes between eRRGs and random genes.

Figure S2. The whole PPI networks. The color of nodes denotes the value of log2FC.

Figure S3. The performance of the trained model 1 (A-E), model 2 (F-J) and model 3 (K-O). Subplot (A/F/K) and (B/G/L) show the ROC curves of the risk score for 1-, 3- and 5-year survival prediction on the training set and test set, respectively. Subplot (D/I/N) and (E/J/O) are the Kaplan-Meier curves showing the difference of overall survival between the high-risk group and low-risk group when training sets and test sets were respectively used. Subplot C, H, and M are the forest plots showing the prognostics signatures.

Figure S4. The calibration plots of the nomograms.

Table S1. The information of the enhancer annotation from Roadmap Epigenomics and ENCODE.

Type of the database	Target of assay	Tissue	Accession
ENCODE	H3K4me1	Stomach	ENCFF994ETY
	H3K27ac		ENCFF787TWQ
Roadmap	H3K4me1		ENCFF375VJL
	H3K27ac		ENCFF722LRM

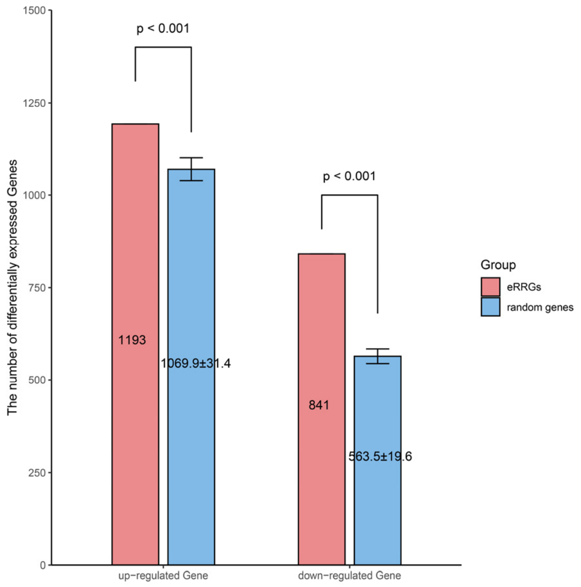

Figure S1. Comparison of the number of differentially expressed genes between eRRGs and random genes.

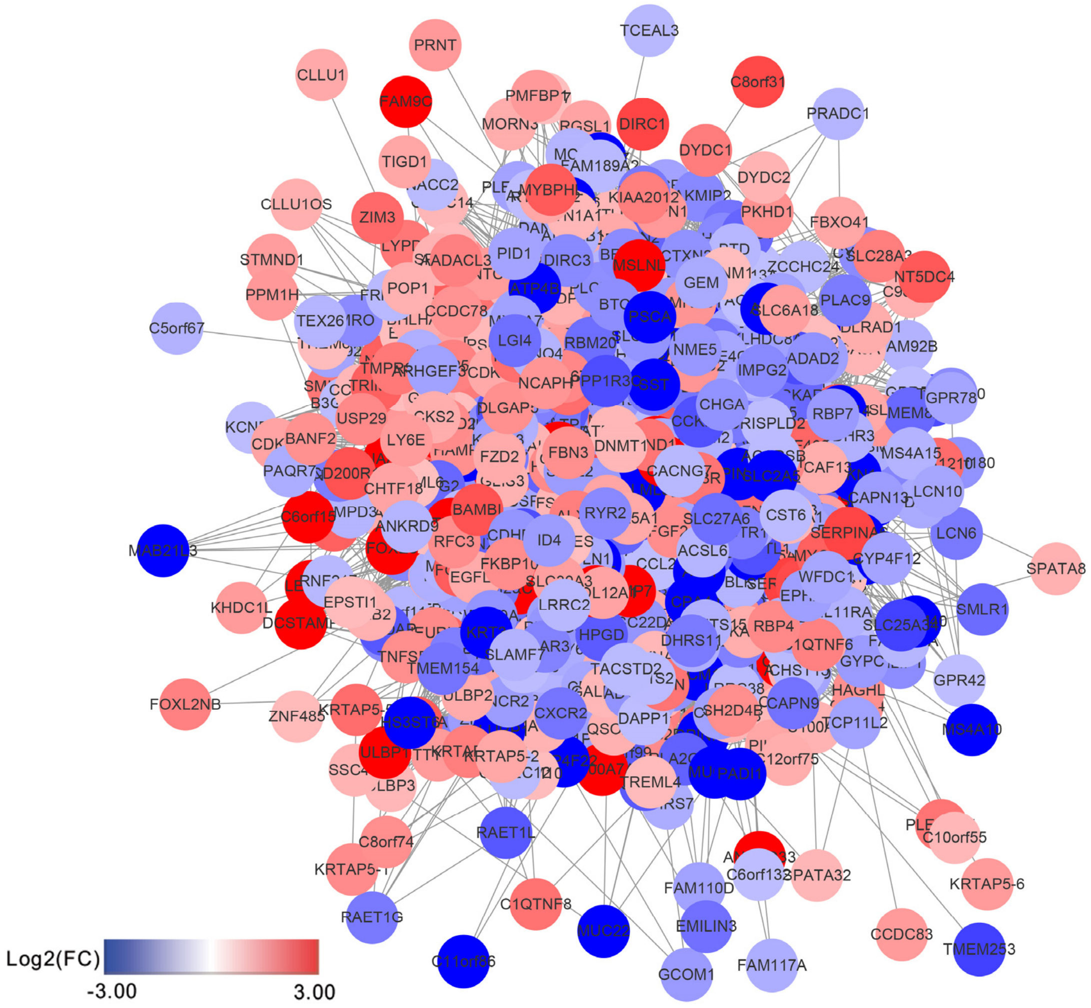

Figure S2. The whole PPI networks.

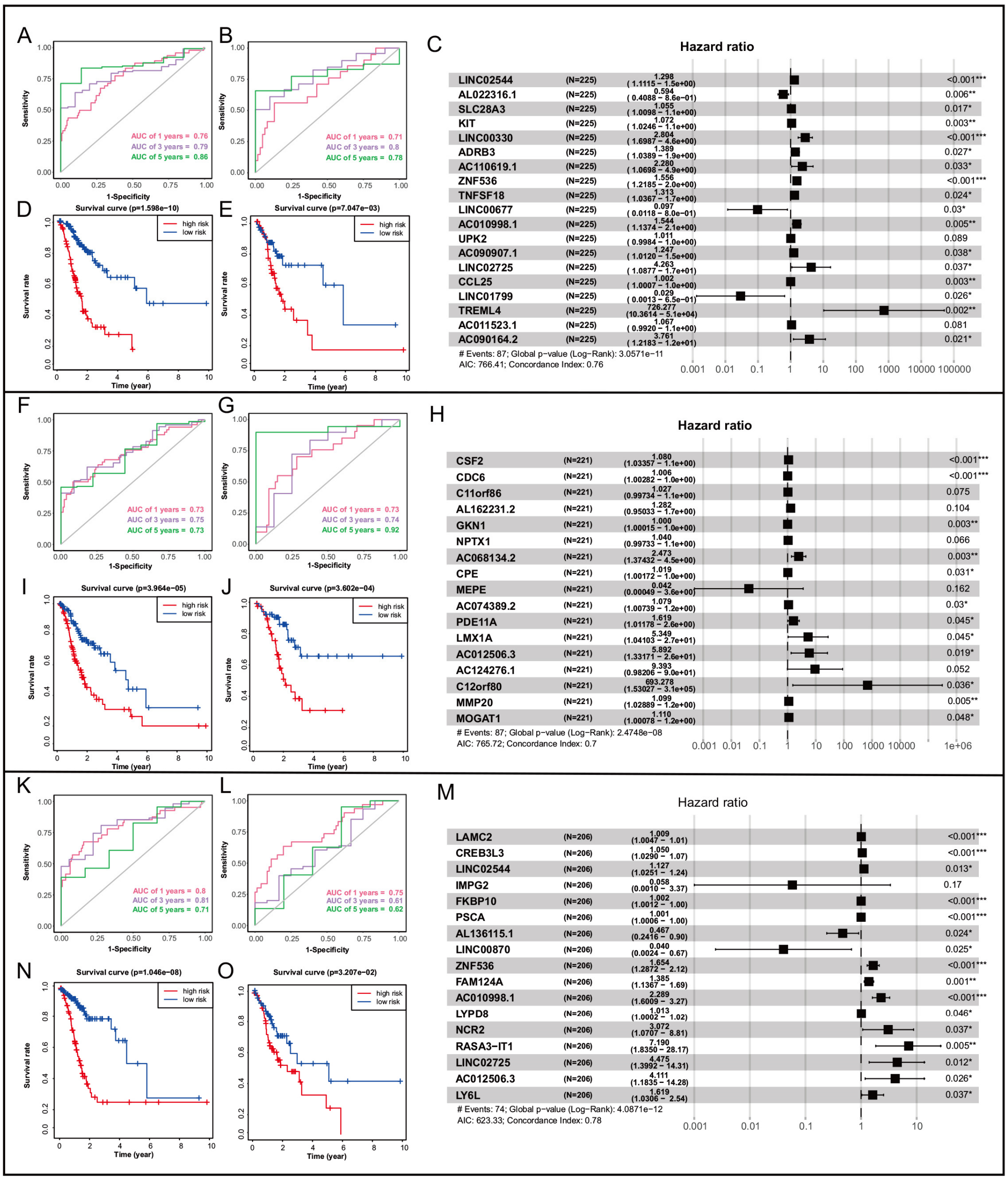

Figure S3. The performance of the trained model 1 (A-E), model 2 (F-J) and model 3 (K-O). Subplot (A/F/K) and (B/G/L) show the ROC curves of the risk score for 1-, 3- and 5-year survival prediction on the training set and test set, respectively. Subplot (D/I/N) and (E/J/O) are the Kaplan-Meier curves showing the difference of overall survival between the high-risk group and low-risk group when training sets and test sets were respectively used. Subplot C, H, and M are the forest plots showing the prognostics signatures.

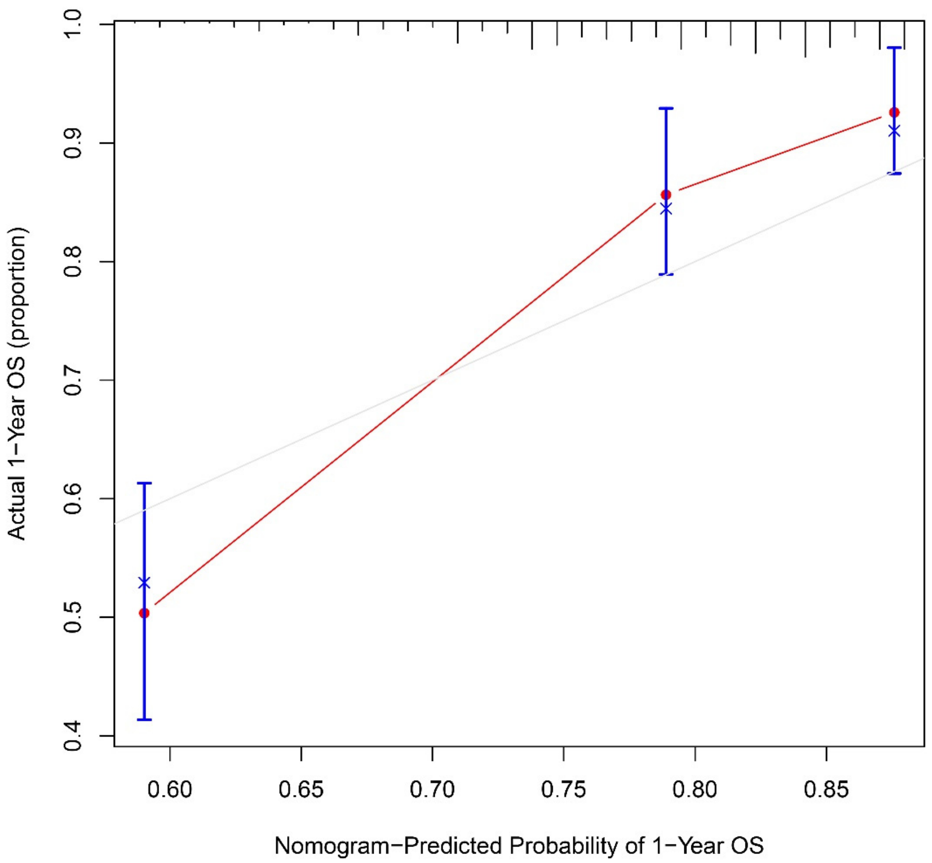

Figure S4. The calibration plots of the nomograms.
